# Update on Lyme Carditis, Groups at High Risk, and Frequency of Associated Sudden Cardiac Death — United States

**Published:** 2014-10-31

**Authors:** Joseph D. Forrester, Jonathan Meiman, Jocelyn Mullins, Randall Nelson, Starr-Hope Ertel, Matt Cartter, Catherine M. Brown, Virginia Lijewski, Elizabeth Schiffman, David Neitzel, Elizabeth R. Daly, Abigail A. Mathewson, Whitney Howe, Lindsay A. Lowe, Natalie R. Kratz, Shereen Semple, P. Bryon Backenson, Jennifer L. White, Phillip M. Kurpiel, Russell Rockwell, Kirsten Waller, Diep Hoang Johnson, Christopher Steward, Brigid Batten, Dianna Blau, Marlene DeLeon-Carnes, Clifton Drew, Atis Muehlenbachs, Jana Ritter, Jeanine Sanders, Sherif R. Zaki, Claudia Molins, Martin Schriefer, Anna Perea, Kiersten Kugeler, Christina Nelson, Alison Hinckley, Paul Mead

**Affiliations:** 1 Epidemic Intelligence Service, CDC; 2 Division of Vector-Borne Infectious Diseases, National Center for Emerging and Zoonotic Infectious Disease, CDC; 3 Wisconsin Department of Health Services; 4 Connecticut Department of Public Health; 5 Massachusetts Department of Public Health; 6 Minnesota Department of Health; 7 New Hampshire Department of Health and Human Services; 8 New Jersey Department of Health; 9 CDC/CSTE applied epidemiology fellow assigned to the New Jersey Department of Health; 10 New York State Department of Health; 11 Pennsylvania Department of Public Health; 12 Division of High-Consequence Pathogens and Pathology, National Center for Emerging and Zoonotic Infectious Disease, CDC

On December 13, 2013, *MMWR* published a report describing three cases of sudden cardiac death associated with Lyme carditis (1). State public health departments and CDC conducted a follow-up investigation to determine 1) whether carditis was disproportionately common among certain demographic groups of patients diagnosed with Lyme disease, 2) the frequency of death among patients diagnosed with Lyme disease and Lyme carditis, and 3) whether any additional deaths potentially attributable to Lyme carditis could be identified. Lyme disease cases are reported to CDC through the Nationally Notifiable Disease Surveillance System; reporting of clinical features, including Lyme carditis, is optional. For surveillance purposes, Lyme carditis is defined as acute second-degree or third-degree atrioventricular conduction block accompanying a diagnosis of Lyme disease. During 2001–2010, a total of 256,373 Lyme disease case reports were submitted to CDC, of which 174,385 (68%) included clinical information. Among these, 1,876 (1.1%) were identified as cases of Lyme carditis. Median age of patients with Lyme carditis was 43 years (range = 1–99 years); 1,209 (65%) of the patients were male, which is disproportionately larger than the male proportion among patients with other clinical manifestations (p<0.001). Of cases with this information available, 69% were diagnosed during the months of June–August, and 42% patients had an accompanying erythema migrans, a characteristic rash. Relative to patients aged 55–59 years, carditis was more common among men aged 20–39 years, women aged 25–29 years, and persons aged ≥75 years (Figure).

To determine the frequency of death among patients with Lyme disease and identify patients in whom carditis might have contributed to death, health officials in seven selected high-incidence Lyme disease states (Connecticut, Massachusetts, Minnesota, New Hampshire, New Jersey, Pennsylvania, and Wisconsin) reviewed convenience samples of cases meeting the surveillance case definition for Lyme disease or Lyme carditis. Patient names were cross-referenced with death certificates to identify patients who died within 1 year of a Lyme disease diagnosis. A suspected case of Lyme carditis–associated mortality was defined as 1) clinically compatible sudden cardiac arrest within 6 months of Lyme disease symptom onset in a person living in, or with recent travel to, a high-incidence Lyme disease area, and 2) detection of antibodies to *Borrelia burgdorferi* in the patient’s serum using two-tier testing criteria.* A confirmed case was defined as a suspected case with pathologic evidence of *B. burgdorferi* infection of the heart confirmed by polymerase chain reaction or by seeing spirochetes in cardiac tissue.

Among 121,894 cases reported during 1995–2013 (120,198 cases with any form of Lyme disease and 1,696 cases with carditis specified), 702 (0.6%) died from all causes within a year of Lyme disease diagnosis. The observed all-cause mortality for these 121,894 patients is below the predicted age-adjusted, all-cause mortality for this population based on national, age-adjusted death rates. Two of these deaths (0.002% of the total) were classified as suspected cases of Lyme carditis–associated mortality after review of available clinical information.

The two suspected cases in this report occurred during June–November in two men in their 40s and 50s. Presenting symptoms included fatigue, malaise, muscle and joint pain, shortness of breath, chest pain, and syncope. Both patients experienced cardiac arrest within 6 weeks of Lyme disease symptom onset. One patient reported erythema migrans, and both had clinical evidence of disseminated infection. Comorbidities included hypertension, diabetes, and hyperlipidemia. Both patients had antibody against *B. burgdorferi* detected by enzyme immunoassay and immunoglobulin M Western blot; one also had antibodies detected by immunoglobulin G Western blot. For neither of these patients was cardiac tissue available for testing.

This report describes the investigation performed by state public health partners and CDC to define high-risk groups and the frequency of death in patients with Lyme carditis. In reported cases, sudden cardiac death remains infrequent when Lyme carditis is recognized and treated with appropriate antibiotic therapy. However, two additional suspected sudden cardiac deaths associated with Lyme carditis were discovered, bringing the total number of cases identified during this investigation to five (three confirmed [1] and two suspected). These cases highlight the public health and clinical challenge that Lyme carditis poses and the need for better primary prevention strategies.

Health care providers should consider Lyme disease as a cause of cardiac symptoms in patients who live in or have visited a high-incidence Lyme disease region, especially during summer and fall months and regardless of whether the patient reports erythema migrans. Additionally, health care providers should investigate the potential for cardiac involvement in patients who have other signs or symptoms of Lyme disease, particularly if they report chest pain, palpitations, lightheadedness, shortness of breath, or syncope. Patients with Lyme carditis should be diagnosed and treated according to current treatment guidelines (3). It is recommended that health care providers remind patients at risk for Lyme disease about common signs and symptoms† and steps they can take to prevent infection.§ Patients who think they might have Lyme disease or Lyme carditis are encouraged to see their health care provider promptly.

## Figures and Tables

**FIGURE:**
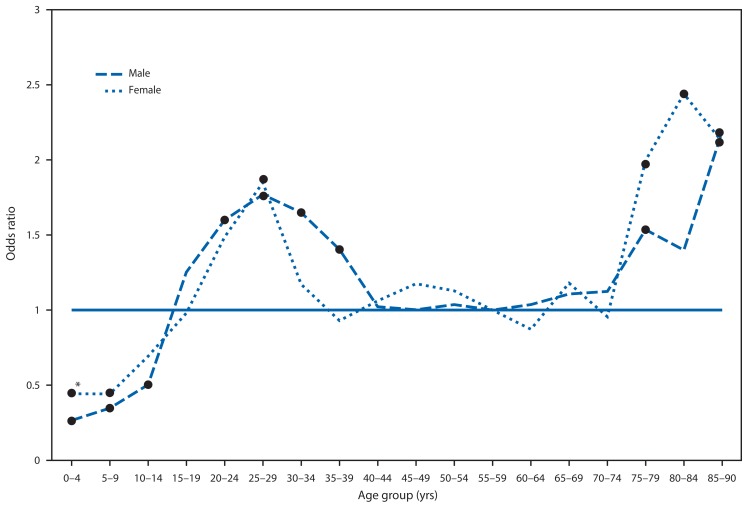
Odds ratios for carditis among Lyme disease patients, by age group and sex — United States, 2001–2010 * Black markers represent statistically significant odds ratios (referent = 55–59 year age group for both sexes).

## Footnotes

* Additional information available at http://www.cdc.gov/lyme/diagnosistesting/labtest/twostep/index.html.

† Additional information available at http://www.cdc.gov/lyme/signs_symptoms/lymecarditis.html.

§ Additional information available at http://www.cdc.gov/lyme/prev/index.html.

## References

[1] CDC (2013). Three sudden cardiac deaths associated with Lyme carditis—United States, November 2012–July 2013. MMWR.

[2] Stanek G, Wormser GP, Gray J, Strle F (2012). Lyme borreliosis. Lancet.

[3] Wormser GP, Dattwyler RJ, Shapiro ED (2006). The clinical assessment, treatment, and prevention of Lyme disease, human granulocytic anaplasmosis, and babesiosis: clinical practice guidelines by the Infectious Diseases Society of America. Clin Infect Dis.

